# Guided post-acceleration of laser-driven ions by a miniature modular structure

**DOI:** 10.1038/ncomms10792

**Published:** 2016-04-18

**Authors:** Satyabrata Kar, Hamad Ahmed, Rajendra Prasad, Mirela Cerchez, Stephanie Brauckmann, Bastian Aurand, Giada Cantono, Prokopis Hadjisolomou, Ciaran L. S. Lewis, Andrea Macchi, Gagik Nersisyan, Alexander P. L. Robinson, Anna M. Schroer, Marco Swantusch, Matt Zepf, Oswald Willi, Marco Borghesi

**Affiliations:** 1 School of Mathematics and Physics, Queen's University Belfast, Belfast BT7 1NN, UK; 2 Institut für Laser-und Plasmaphysik, Heinrich-Heine-Universität, Düsseldorf D-40225, Germany; 3 Department of Physics E. Fermi, Largo B. Pontecorvo 3, Pisa 56127, Italy; 4 Consiglio Nazionale delle Ricerche, Istituto Nazionale di Ottica, Research Unit Adriano Gozzini, via G. Moruzzi 1, Pisa 56124, Italy; 5 Central Laser Facility, Rutherford Appleton Laboratory, Didcot, Oxfordshire OX11 0QX, UK; 6 Helmholtz Institut Jena, 07743 Jena, Germany; 7 Institut für Optik und Quantenelektronik, Universität Jena, 07743 Jena, Germany

## Abstract

All-optical approaches to particle acceleration are currently attracting a significant research effort internationally. Although characterized by exceptional transverse and longitudinal emittance, laser-driven ion beams currently have limitations in terms of peak ion energy, bandwidth of the energy spectrum and beam divergence. Here we introduce the concept of a versatile, miniature linear accelerating module, which, by employing laser-excited electromagnetic pulses directed along a helical path surrounding the laser-accelerated ion beams, addresses these shortcomings simultaneously. In a proof-of-principle experiment on a university-scale system, we demonstrate post-acceleration of laser-driven protons from a flat foil at a rate of 0.5 GeV m^−1^, already beyond what can be sustained by conventional accelerator technologies, with dynamic beam collimation and energy selection. These results open up new opportunities for the development of extremely compact and cost-effective ion accelerators for both established and innovative applications.

## Abstract (web summary)

Intense laser-driven acceleration mechanisms are promising for the realization of compact particle accelerators. Here, the authors present a miniature linear accelerating module for laser-driven protons from a foil that addresses limitation in terms of peak energy, bandwidth and beam divergence.

## Graphical abstract

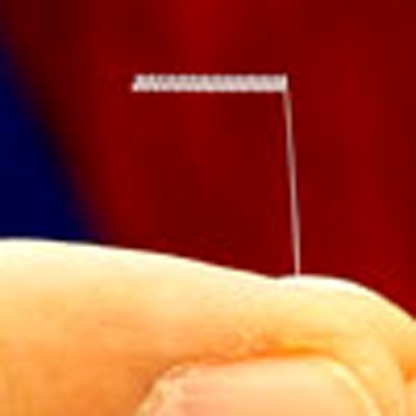

In the context of developing compact, high current ion accelerators, the study of laser-driven acceleration mechanisms and the characterization and optimization of the ion beams produced, have been, over the past decade, very active areas of research^1^,^2^,^3^,^4^. Most experimental studies so far have dealt with the so-called target normal sheath acceleration (TNSA) mechanism, where ions are accelerated by space charge fields set up by relativistic electrons at the target surfaces. Despite the remarkable beam emittance and brightness, the inherent divergence and broad exponential energy spectrum of the TNSA beams limit their applicability in wide range of sectors^4^, such as healthcare, industry, nuclear physics, high-energy density physics. Furthermore, reaching high-energies (>100s MeV) as required by important accelerator applications (for instance, clinical proton therapy^5^,^6^,^7^), will require, according to the current understanding of the acceleration mechanism^2^,^3^, significantly larger laser systems than affordable in many cases. Coupling laser-driven ions to conventional RF stages for post-acceleration and beam control^8^ is an approach currently being explored, which however, is inherently less attractive than an all-optical approach in terms of both cost and compactness.

In this paper, we present a laser-driven miniature device for simultaneous energy selection, collimation and post-acceleration of ions, which is an uniquely attractive combination compared with the methods explored so far for improving the quality of laser-driven ion beams^9^,^10^,^11^,^12^,^13^,^14^,^15^. The scheme exploits ultra-short (10s of picoseconds), high-amplitude, unipolar electromagnetic (EM) pulses generated in the interaction of a high-power laser with a solid target^16^,^17^. The technique is demonstrated in a proof-of-principle experiment using a 200 TW university-scale laser, achieving post-acceleration of protons to enhance their energy by ∼5 MeV over less than a centimetre of propagation, that is, an accelerating gradient ∼0.5 GeV m^−1^. This is already beyond what can be sustained by conventional accelerator technologies^18^,^19^,^20^ and can be enhanced significantly at higher laser intensities.

## Results

### Experimental setup

The experiment was carried out using the ARCTURUS laser system at Heinrich-Heine-Universität, Düsseldorf, Germany. Laser pulses of ∼30 fs duration and energy ∼3 J were focused on thin metallic foils by an *f*/2 off-axis parabola to a spot of ∼4 μm full-width at half-maximum, delivering peak intensities of a few times 10^20^ W cm^−2^. A stack of radiochromic film (RCF) detectors was used to diagnose the spatial and spectral distribution of the proton beam. The stacks of RCF were placed at 35 mm from the proton-generating foils. The RCF dose response was absolutely calibrated against batches of RCFs exposed to different known proton doses from a particle accelerator^21^.

**Figure 1:**
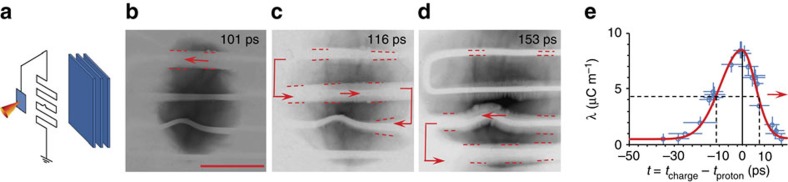
Proton probing of EM pulse. (**a**) Schematic representation of the setup for diagnosing the EM pulse propagation along a folded wire. A small bent was made in one of the wire segments to act as a fiducial. The corresponding proton radiographs obtained at three different times (as labelled on each image) are shown in **b**–**d**. In these images, the film darkness is proportional to the proton flux. The red arrows in the images indicate the direction of charge flow in the folded wire pattern. The red dotted lines are eye guides for the width of the proton deflected region. The scale bar (solid red line) shown in **b** refers to 1 mm in the plane of the folded wire pattern. (**e**) Temporal profile of the positive charge pulse travelling along the folded wire. The *x* axis of the graph corresponds to the relative probing time, *t*=*t*_charge_−*t*_proton_, where *t*_proton_ is the probing time of a given point on the folded wire by the protons reaching the Bragg peak in the given RCF layer, and *t*_charge_ is the time of arrival of the peak of the charge pulse at that point. The experimental uncertainty in time is determined by the transit time of protons through the electric field region and the energy resolution of the active layers of RCF (ref. 25). The uncertainty in the charge density is estimated from the uncertainty in measuring the width of the proton deflection from the RCF data. The red arrow in the graph indicates the direction of propagation of the pulse.

**Figure 2:**
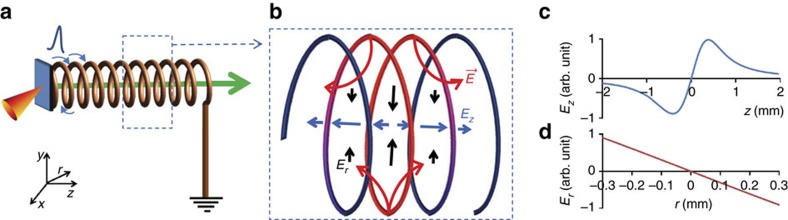
Helical coil working principle. (**a**) Schematic representation of the target designed for optimizing the beam parameters of laser-driven protons. In this configuration a helical coil, made of a metallic wire, is attached to the laser-irradiated thin foil at one end and grounded at the other end. The helical coil design guides the EM pulse carrying the neutralizing charge around the proton-beam axis and allows synchronizing its longitudinal propagation (that is, along *z*) with protons having a given energy within the beam. (**b**) Schematic representation snapshot showing the electric field configuration inside the coil. The red section of the coil represents the segment charged by the travelling pulse at a given moment of time, where the red arrows represent the electric field (**E**) lines originating from the coil, the black and blue arrows represent radial (*E*_r_) and longitudinal (*E*_z_) components of the electric field, respectively. The length of the black and blue arrows represents the relative strength of the field at different locations. (**c**,**d**) *E*_z_|_(*r*=0)_ and *E*_r_|_(*z*=0)_ profiles inside the coil at a given time, where *z*=0 corresponds to the location of the peak of charge density along the coil at that time. The field profiles are calculated by the subroutine that defines the input electric field configuration for particle tracing in the PTRACE simulation (see Methods). Dynamics are modelled using an asymmetric Gaussian pulse profile of 5 ps rise and 10 ps decay, as obtained in the experiment shown in Fig. 1e, travelling along a helical coil with the same dimensions as the one used in the experiment illustrated in Fig. 3.

Data presented in this paper were collected by using two different target geometries. The propagation of the ultra-short, high-amplitude EM pulse along a thin wire connected to the laser-irradiated target was initially characterized by using the target geometry shown by the schematic representation in Fig. 1a. In this case, the TNSA proton beam generated at the rear of the laser-irradiated target was used as a particle probe in a point-projection arrangement to obtain time resolved snapshots (see Methods section for additional information) of the pulse propagation along the wire (0.1 mm diameter Al wire), which was folded to a square wave pattern in front of and parallel to the interaction foil. The folded wire design was chosen to enable us to follow, in the diagnostic field of view, the pulse propagation along the wire over an extended distance (up to ∼35 mm). The second part of the experiment, which demonstrates the technique of using the EM pulse for control and optimization of the proton-beam parameters, was carried out by using a target that comprises a helical coil of suitable dimensions connected to the rear side of the laser irradiated foil, as shown in the Fig. 2a. The details of the target geometries and dimensions are discussed in the respective sections and figure captions.

### Experimental results

*Generation of ultra-short EM pulse*. At relativistic intensities (>10^18^ W cm^−2^) the interaction produces a large number of ‘hot' (MeV) electrons^22^,^23^. A small fraction of the hot electron population escapes from the target charging it rapidly to MV potential^9^,^10^,^24^. The sudden charge separation leads to the generation of a strong EM pulse (with an electric field amplitude of GV m^−1^ order of magnitude, depending on laser intensity), which travels along the surface of the target at approximately the speed of light^16^,^17^. If the charged-up target is connected to the ground via a support wire, the pulse effectively contributes to driving a neutralizing current—in a situation analogous to a charged capacitor being suddenly connected to a transmission line. The high temporal resolution of the proton radiography technique^16^,^25^,^26^,^27^ employed in our experiment allowed visualizing the propagation of the pulse along a thin metallic wire attached to the target. The proton radiograph of Fig. 1b shows the presence of an outwardly directed, strong electric field (of the order of GV m^−1^) around the top segment of the folded wire, causing a clear deflection of the probe protons normal to the wire, while the bottom segments remain electrically neutral. At later times, the electric field region is seen to be moving downwards along the wire, while leaving the top segment electrically neutral (Fig. 1d). This confirms the localization in space of the electrically charged region at any given time. By following the proton deflection across the folded wire geometry as observed in different layers of the RCF stack, a temporal profile of the propagating charge pulse was reconstructed with the help of particle-tracing simulations, employing the PTRACE code (see the Methods section). The temporal profile of the charge pulse is shown in Fig. 1e having rise and decay times of ∼5 and ∼10 ps, respectively. The velocity of the pulse flowing down the wire is measured as *v*=(0.96±0.04)*c*, where *c* is the speed of light in vacuum. The total charge contained within the pulse is estimated (by integrating the linear charge-density profile *λ*(*t*) in Fig. 1e) as ∼60 nC, which agrees with an order of magnitude estimation of the net charge left in the target because of the escaping hot electrons^9^,^24^,^25^. The temporal profile of the neutralizing current pulse can be obtained by multiplying *λ*(*t*) by its velocity *v*, which estimates a peak current of the order of kiloamp for the case shown in Fig. 1e.

*Laser-driven travelling-wave ion accelerator*. The localized, ultra-high electric field associated with the travelling EM pulse can be employed for controlling and optimizing bursts of laser-accelerated MeV ions on a picosecond timescale. By directing the EM pulse along a helical path around the proton-beam axis (see Fig. 2a), one can synchronize, over an extended distance, the longitudinal propagation of the electric field (associated with the pulse) with the transiting protons within a given energy range. A sketch of the electric field configuration inside the coil geometry is shown schematically in Fig. 2b. The calculated longitudinal (*E*_z_) and radial (*E*_r_) electric field profiles inside the coil due to the pulse are shown in Fig. 2c,d. At a given time, the pulse spreads over approximately two windings of the coil used in Fig. 2b, and therefore the profiles are qualitatively similar to those produced by a uniformly charged ring^28^ giving, for example, for the longitudinal component, *E*_z_(*z*)|_(*r*=0)_=2*πkλRz*/(*R*^2^+*z*^2^)^3/2^, where *k* is the Coulomb's constant and *R* is the radius of the ring. The radial and longitudinal components of the moving electric field act, respectively, towards focussing and acceleration (or deceleration, depending on the position of protons with respect to the charged segment of the coil) of the protons synchronous with the EM pulse. Conversely, the unsynchronized protons maintain their intrinsic divergence, and can be discarded with an appropriate spatial selection.

**Figure 3:**
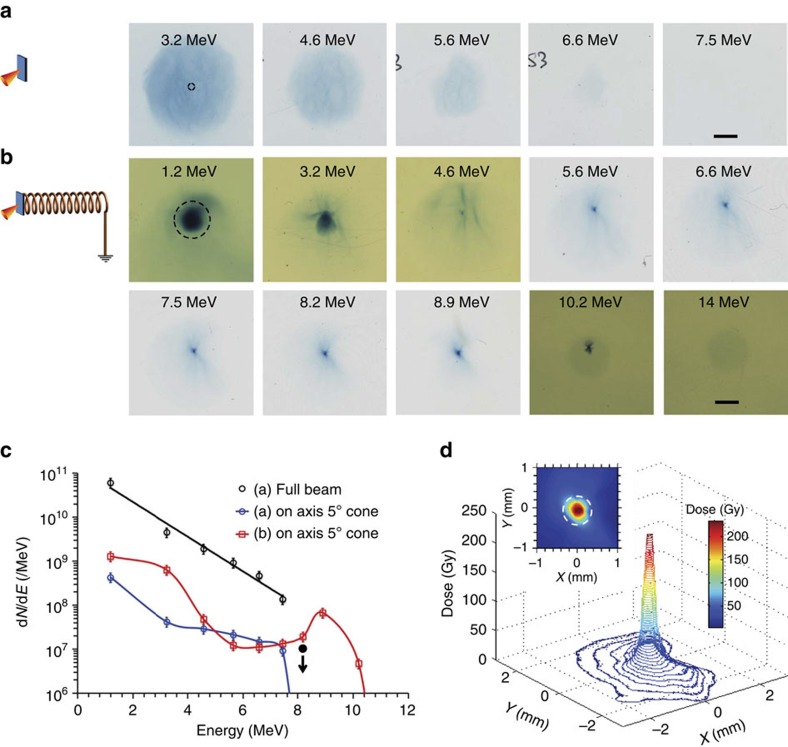
Effect of helical coil on TNSA beam. In comparison with the typical proton-beam profile obtained from a flat-foil target in the experiment shown in (**a**), (**b**) shows the beam profile obtained from the helical coil target. Au foils (10 μm thick) were used as the laser interaction target in both cases. The coil was made of 100 μm aluminium wire and had internal diameter, pitch and length of 0.7, ∼0.28 and 8.7 mm, respectively. The RCF stack was placed at 35 mm from the target. RCF images of 50 mm × 50 mm size are shown in **a**, which are five times larger compared with the RCF images shown in **b** (the black scale bar on the last piece of RCF in **a** and **b** correspond to 10 and 2 mm respectively), in order to account for the large divergence of the TNSA beam produced from the flat foils. The black-dashed circles on the first RCF layer for both **a** and **b** correspond to the projection of the exit ring of the coil on the RCF (∼2.8 mm diameter circle corresponding to ∼5° full cone angle). (**c**) shows the comparison between proton spectra from the reference flat-foil target and the helical coil target shown in **b** obtained by spectral deconvolution of the RCF signals described in refs 37, 38. The error bars were estimated from the possible error in dose conversion^21^ and uncertainties in background substraction. Since the flat-foil proton signal at ∼8 MeV was below the detection threshold, the solid black circle shows the upper bound for proton signal calculated by considering the detection threshold of the RCF (∼10^5^ protons per MeV mm^−2^) and an overly generous beam size (10 mm diameter on the RCF, which is similar to the beam size at 6.6 MeV shown in **a**) for protons at that energy. (**d**) Three-dimensional profile of the pencil beam of protons obtained in the RCF corresponding to ∼9 MeV, where the inset shows the two-dimensional dose map of the central part of the beam. The white dashed circle in the insert represents the internal diameter of the helical coil.

Figure 3 shows proof-of-principle results using the helical coil target. In contrast to the typical divergent proton beam produced by a flat foil (Fig. 3a), the helical coil target (Fig. 3b) produced a highly collimated beam of protons of energy significantly higher than from a flat foil, as clearly observed in the RCF stack diagnostic. In the flat-foil proton spectrum shown in Fig. 3c, the proton number after 7 MeV falls below the detection threshold of the RCF—one can estimate 10^7^ protons per MeV as an absolute maximum for 8 MeV protons, while assuming a very generous beam diameter (see caption of Fig. 3 for details). On the other hand, a spectral peak at ∼9 MeV (with ∼10^8^ protons per MeV at the peak), with detectable RCF signal up to 10 MeV is observed in the case of Fig. 3b, providing a clear indication of an increase in proton energy resulting from the helical coil. Furthermore, as shown in Fig. 3d, the diameter of the focussed, narrow-band, 9 MeV beam at 35 mm from the target is less than the internal diameter of the coil, which indicates nearly collimated (<0.5° divergence) propagation of the protons after exiting the coil structure.

**Figure 4:**
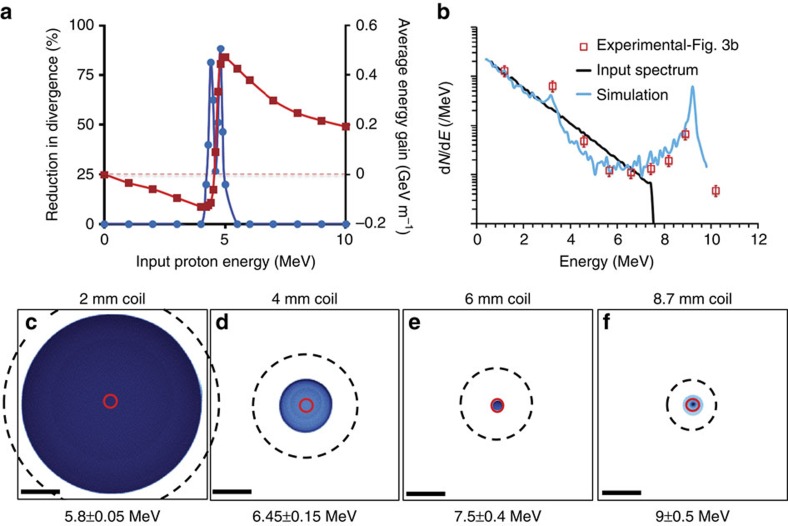
Simulations of focusing and post-acceleration by the helical coil. (**a**) Reduction in beam divergence (filled blue circles) and the gain in energy (filled red squares) for different input proton energies as obtained from simulations carried out for the case shown in Fig. 3b. (**b**) Comparison between experimental and simulated proton spectra at the detector plane for the case shown in Fig. 3b. An exponential input spectrum for protons (as shown), similar to the one obtained from the reference flat foil, was used in the simulation. (**c**–**f**) Simulated spatial profiles of the proton beam at the detector plane produced by different lengths of the helical coil target shown in the case of Fig. 3b. The black scale bars at the bottom left of the images correspond to 2 mm on the RCF plane. A divergent beam of 4.8 MeV protons was used as input in the simulations, which experience maximum accelerating field according to the graph shown in **a**. The output beam energy in each case is mentioned on the images. The black-dashed circle represents the projection of the exit ring of the coil on the RCF and the red solid circle shows the internal diameter of the coil.

Particle-tracing simulations (see Methods for more details) carried out using the target parameters and the measured charge pulse characteristics (amplitude, temporal profile and speed) show that in the case of Fig. 3b the pulse is optimally synchronized for input energy 4–5 MeV (see Fig. 4a). While the radial component of the associated field acts on constraining the divergence of these protons, the longitudinal component of the electric field progressively accelerates the leading part of the synchronized proton bunch at a rate close to 0.5 GeV m^−1^, as shown in Fig. 4c–f. The analogy with a uniformly charged ring model yields an order of magnitude agreement: the longitudinal field has a peak amplitude 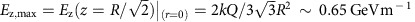, while using *Q* (total charge in the ring)=30 nC (accounting for the charge spreading over two windings) and *R*=400 μm. As expected, protons in the trailing part of the synchronized bunch experience deceleration during their travel. The protons which are co-propagating with the central portion of the pulse experience virtually no longitudinal field and their energy is unchanged, while they are focused much more strongly, and have already diverged by the time they reach the RCF detector. The simulated proton spectrum agrees well with the experimental data points as shown in Fig. 4b, while using an input proton spectrum mimicking the experimental data from flat foils. The two peaks in the simulated proton spectrum on either side of 5 MeV are due to the simulated energy gain/loss shown in Fig. 4a. Considering that the 10^8^ protons per MeV at the spectral peak, observed in the experimental data, are produced by the post-acceleration of ∼5 MeV protons, Fig. 3c suggests that gradual focussing of synchronous protons along the coil resulted in an overall collection efficiency of 7%, which is three times better than what would be captured, for an undeflected beam, within the solid angle sustained by the exit ring of the coil.

## Discussion

**Figure 5:**
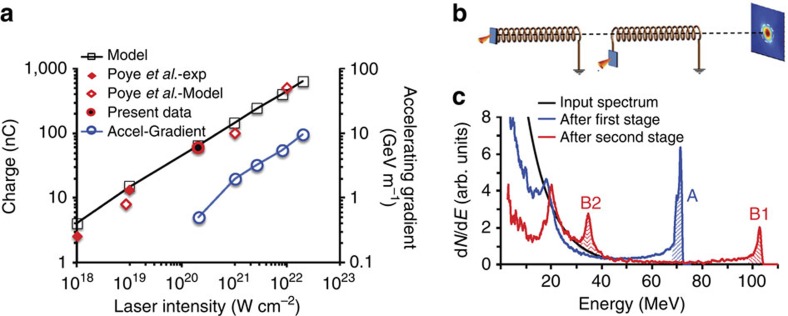
Scaling to higher laser intensity and multistage acceleration. (**a**) Total charge carried by the pulse moving along the wire connected to the laser-irradiated target, plotted against incident laser intensity on target (black solid line). The blue solid line shows the accelerating gradient inside a helical coil of same diameter and pitch as the one used in our experiment. (**b**) Schematic representation of a double-stage acceleration setup using two helical coils driven by two laser pulses. (**c**) Comparison between the simulated proton spectra, taken at 50 mm from the proton source over a 2 mm × 2 mm area, obtained for: flat-foil proton source (black-input spectrum in the simulation), single-stage (blue) and double-stage (red) coil re-acceleration. Both coils were of 0.5 mm internal diameter and 10 mm long, with variable pitch suited to their input proton energies (∼40 and ∼70 MeV, respectively, for the first and second stages). The parameters for the charge pulse in both coils were taken as those expected from the interaction of a PW, 30 fs laser (e.g. as available at GIST, Korea^33^) with a thin target, leading to an acceleration gradient of ∼3 GeV m^−1^ (discussed in the text). The shaded areas A, B1 and B2 represent, respectively, the proton bunch accelerated from the first coil and the proton bunches accelerated and decelerated by the second coil. The total number of particles in the bunches B1 and B2 is approximately equal to the number of particles in the bunch A. The time of arrival of the charge pulse at the entrance of the second coil was synchronized with the arrival of the ∼70 MeV proton bunch produced by the first coil.

While the experiment shows a proof-of-principle demonstration employing a university-scale laser system, there is significant scope for further development of the technique using higher-intensity lasers and more refined target arrangements. For a given diameter of the helical coil target, the accelerating field inside the coil structure, as discussed above, is directly proportional to the charge (*Q*=∫*λ*(*l*)d*l*) contained in the pulse, which is equal to the number of electrons (*N*_esc_) escaping from the laser-irradiated target during the interaction times the electron charge (*e*). The scaling for *eN*_esc_(=*Q*) with incident laser intensity can be obtained by using the simple phenomenological model described by Kar *et al.*^9^ Figure 5a shows estimates of *Q* obtained using this model, for constant laser pulse length (30 fs) and focal spot size on the target (4 μm). A prudent 30% laser-electron conversion efficiency is assumed in this model, which may be an underestimate for intensities above 10^20^ W cm^−2^ (refs 22, 29, 30, 31, 32). In any case, as can be seen in this figure, the scaling agrees well not only with our experimental data but also with the experimental data and model reported recently by Poye *et al.*^24^ for similar pulse duration and focussing conditions.

The increase in the accelerating gradient with the incident laser intensity is shown in Fig. 5a. This was estimated from PTRACE simulations while considering the same helical coil geometry as used in our experiment and the peak charge density calculated from the estimated *Q* for a given laser intensity. Considering for instance the parameters of the PW laser recently commissioned at GIST, Korea^33^, the energy gain per unit length for a helical coil (0.5 mm internal diameter and 10 mm long) would be 3 GeV m^−1^. As shown in Fig. 5b, PTRACE simulations suggest that injecting an exponential spectrum of protons with ∼40 MeV cutoff (as produced on such a laser system^34^) into the coil, one would expect a narrow energy bandwidth pencil beam of ∼70 MeV at the coil output. The pitch of the coil employed in this simulation varied along its length in order to keep the charge flow synchronized with the selected proton bunch as it is being accelerated. This approach can, in principle, be extended to maintain an efficient acceleration over coils of several cm length.

Furthermore, since the helical coil device is effectively a separate module with respect to the primary ion acceleration process producing the seed beam which enters the coil, one can envisage a multistage implementation of the process, employing sequential, multiple coils irradiated by separate, appropriately timed laser pulses—a scheme with high promise for the development of a compact linear particle accelerator. As shown in the insert of Fig. 3d, the diameter of the proton beam at 35 mm from the target was smaller than the internal diameter of the coil, which implies that the output beam is quasi-collimated. Therefore, it would be possible to feed the entire output beam from one stage into a second coil of similar (or even smaller) diameter, placed a few mm after the previous coil. As an example, Fig. 5c shows the simulated proton spectrum obtained from a two stage configuration (as shown by a schematic representation in Fig. 5b). The bunch of protons accelerated to ∼70 MeV by a single-stage coil can be re-accelerated to higher energies by employing suitable coil and charge pulse parameters. It should also be noted that, using a similar arrangement, a separately driven coil could be used for post-acceleration of ion beams generated by any alternative acceleration mechanism^4^ on a primary target.

In conclusion, a technique to achieve simultaneous focussing, energy selection and post-acceleration of laser-driven ions is reported, which is based on harnessing, in a travelling wave accelerator arrangement, the fields arising from ultra-short, large-amplitude EM pulses generated by intense lasers. An accelerating gradient of ∼0.5 GeV m^−1^ is demonstrated by the technique in a proof-of-principle experiment using a 200 TW laser. This development sets a cornerstone for next-generation, extremely compact and cost-effective proton accelerators, which complements the current drive for miniaturization in advanced accelerator technology.

## Methods

### Proton radiography

In a typical arrangement employing broadband TNSA protons, proton radiography^26^,^27^ provides, in a single-shot, multiple-snapshots (corresponding to sequential time frames) of the probed region. Instead of using a second laser beam to generate the probe protons, in this experiment we used a self-imaging arrangement, where both the probe protons and the EM pulse were generated from the same laser interaction. Protons generated from the rear surface of the target foil were used to probe the propagation of the EM pulse along a folded wire segment, as shown in Fig. 1a. The presence of an electric field in the probed region is detected via the modulation in the proton flux distribution induced by local particle deflections.

### PTRACE simulation

The particle-tracing simulations presented in this paper were performed using the PTRACE code (ref. 35). This code simulates in three dimensions the propagation of protons from the source to the detector through the probed region consisting of electric and magnetic fields. At the core of the code, the equation of motion is computed by a Runge–Kutta fourth-order algorithm coupled with an adaptive step size monitoring routine.

The linear charge density at a given time and location on the folded wire segment, shown in Fig. 1, was obtained by matching the experimental proton deflection with the deflection produced by PTRACE for the corresponding probe proton energy. Using the charge-density profile as a template, a set of dynamic PTRACE simulations were carried out in order to evaluate the transit time effect of the probe protons towards the rise time, decay time and peak charge density estimated from the static simulations. We assumed the EM pulse to propagate as a transverse electro-magnetic (TEM) mode^17^ and the electric field **E** around the wire was calculated numerically from the linear charge-density profile shown in Fig. 1e. The dynamic simulation was set up by allowing a given positive charge pulse to travel along the folded wire structure with the same dimensions as in the experiment. The electric field at any given point ‘P', experienced by a transiting proton at a given time, was computed by adding electric field vectors at ‘P' due to every element of the wire, while considering the position of the pulse in the wire at that probing time. The best match was obtained for a charge pulse profile similar (within the diagnostic's temporal resolution of a few ps) to that obtained from the PTRACE simulations.

The helical coil was modelled in PTRACE using a cylindrical coordinate system. Using the same physical parameters for the coil as used in the experiment, a charge pulse of a given temporal profile was allowed to travel along it. The electric field at any given point at a given time was computed numerically by the method mention above. The proton source in the code was modelled as a point source located at the centre of the entrance plane of the coil, emitting protons towards the coil with a given energy spectrum and divergence. After the transit of the protons through the field region defined by the coil, the PTRACE renderer calculates the response of the stack detector for every incident proton. Energy deposited in each layer of the stack is computed by using the stopping range of protons in the RCF plastic calculated by SRIM (ref. 36) simulation.

## Additional information

**How to cite this article**: Kar, S. *et al.* Guided post-acceleration of laser driven ions by a miniature modular structure. *Nat. Commun.* 7:10792 doi: 10.1038/ncomms10792 (2016).
